# Supplementary material for: Multiple drainage reversal episodes and glacial refugia in a Patagonian fish revealed by sequenced microsatellites
Source: Proc Biol Sci. 2020 Jun 3;287(1928):20200468. doi: 10.1098/rspb.2020.0468 (PMC7341911; doi:10.1098/rspb.2020.0468)
Supplement: Microsatellite Primer sequences and AMOVA tables [file rspb20200468supp1.docx]

**ELECTRONIC SUPPLEMENTARY MATERIAL**

**Multiple drainage reversal episodes and glacial refugia in a Patagonian fish revealed by sequenced microsatellites**

Daniel E. Ruzzante^1^, Annie P. Simons^1^, Gregory R. McCracken^1^, Evelyn Habit^2^, Sandra J. Walde^1^

1: Department of Biology, Dalhousie University, Halifax, Nova Scotia, Canada B3H4R2

2: Laboratorio de Ecología y Conservación de Peces, Departamento de Sistemas Acuáticos, Facultad de Ciencias Ambientales y Centro EULA, Universidad de Concepción. Concepción, Chile

Correspondence Daniel E. Ruzzante, Department of Biology, Dalhousie University, Halifax, NS B3H4R2, Canada. Email: [daniel.ruzzante@dal.ca](mailto:daniel.ruzzante@dal.ca)

***Primer Design and Testing***

A single DNA sample, chosen for quality and quantity of DNA, was used for the microsatellite loci development/primer design process. We followed the methods by McCracken *et al.* found in supplement MOESM3 in [1]. Initially, approximately 40500 microsatellite loci/primer pairs were detected using Msatcommander 1.0.8 [2] which satisfied our criteria (product size between 50-140bp, and a minimum of 6 repeats). We selected 75 microsatellite loci/primer combinations for further testing, of which 45 consisted of dinucleotide repeat motifs, and the remaining 30 were trinucleotide repeat motifs. These selected primer were then tailed with Illumina (San Diego CA, USA) Read1 __(CCCTACACGACGCTCTTCCGATCT)_ and Read2 __(GTTCAGACGTGTGCTCTTCCGATCT)_ sequencing primers and ordered from DNA technologies (IDT, Coralville, IA, USA).

96 individuals from various locations were chosen for testing to maximize the amount of polymorphism detected. The 75 chosen loci were amplified using 2 multiplex reactions with 38 and 37 primer pairs respectively. These multiplex PCRs were conducted then diluted, indexed, cleaned and sequenced on an Illumina MiSeq Benchtop Sequencer (Illumina, San Diego, CA, USA) following the methods outlined in [3] (supporting information).

References

1. Yu D, Ding X, Zhang Z, Zeng Y, Liu H, Wang L, … Zhang Q. 2018 Correction to: Microsatellite records for volume 10, issue 2. *Con. Gen. Res.*, 1–2. https://doi.org/10.1007/s12686-018-1056-6
2. [Faircloth BC](https://www.ncbi.nlm.nih.gov/pubmed/?term=Faircloth%20BC%5BAuthor%5D&cauthor=true&cauthor_uid=21585724). 2008 msatcommander: detection of microsatellite repeat arrays and automated, locus-specific primer design. [*Mol. Ecol. Res.*](https://www.ncbi.nlm.nih.gov/pubmed/21585724) **8**(1), 92-94.
3. Ruzzante DE, McCracken GR, Salisbury SJ, Brewis H, Keefe D, Gaggiotti OE, Perry R. 2019 Landscape, colonization and life history: Their effects on genetic diversity in four sympatric species inhabiting a dendritic system. *CJFAS* **76,** 2288-2302.

Table S2. Sample information for all 46 collections from 16 river systems. **#**: Sample number. **Drainage:** indicates whether the system drains into the Pacific (PAC) or Atlantic (ATL) Ocean. **HW:** indicates whether the headwaters are west of the highest Andean peaks in Chile (CH) or east of them in Argentina (AR). Five drainages are trans-Andean, i.e., their headwaters are east of the highest Andean peaks in Argentina, but they drain through the valleys across the mountains into the Pacific Ocean. These are the Valdivia, Puelo, Futalaufquen/Yelcho, Baker, and Pascua systems. Note that there are no trans-Andean systems with headwaters west of the Andes that drain east, across the mountains into the Atlantic. **Sampling location**: the lake or river where samples were collected from; (CH) or (AR) indicates whether the location is in Chile or Argentina. **ID**: sample label. **N:** sample size. **Lat**. and **Long**: Latitude and Longitude in decimal degrees. **Collection Date**: Month and year of sample collection.

| # | Drainage | HW | River system | Sampling Location | ID | N | Lat. (^o^S) | Long. (^o^W) | Collection Date |
| --- | --- | --- | --- | --- | --- | --- | --- | --- | --- |
| 1 | PAC | CH | Maipo | Estero El Belloto (CH) | RMAIP | 13 | -33.0972 | -71.10858 | Dec-06 |
| 2 | PAC | CH | Nilahue | Estero Nilahue (CH) | RNILA | 25 | -34.4835 | -72.01623 | Jan-07 |
| 3 | PAC | CH | Rapel | Río Tinguiririca (CH) | RTING | 27 | -34.6115 | -71.00131 | May-07 |
| 4 | PAC | CH | Biobío | Río Biobío (CH) | RBIOB | 29 | -37.5659 | -72.4898 | Aug-04 |
| 5 |  |  |  | Río Ramadillas (CH) | RRAMD | 1 | -37.3081 | -73.26449 | Mar-07 |
| 6 | PAC | AR | Valdivia* | Río Cruces (CH) | RCRUC | 2 | -39.5438 | -72.95889 | Apr-07 |
| 7 |  |  |  | Lago Panguipulli (CH) | LPANG | 25 | -39.6446 | -72.32338 | Mar-06 |
| 8 |  |  |  | Lago Neltume (CH) | LNELT | 7 | -39.8099 | -71.9945 | Mar-06 |
| 9 | PAC | CH | Bueno | Río Bueno (CH) | RBUEN | 31 | -40.3474 | -73.17888 | Mar-06 |
| 10 |  |  |  | Río Rahue (CH) | RRAHU | 4 | -40.6715 | -73.12497 | Mar-07 |
| 11 |  |  |  | Lago Puyehue (CH) | LPUYE | 13 | -40.6909 | -72.54541 | Mar-06 |
| 12 |  |  |  | Lago Rupanco (CH) | LRUPA | 15 | -40.7897 | -72.68628 | Apr-06 |
| 13 | PAC | CH | Maullín | Río Pescado (CH) | RPESC | 27 | -41.2807 | -72.85311 | Mar-08 |
| 14 |  |  |  | Río Petrohue (CH) | RPETR | 29 | -41.3782 | -72.31113 | Apr-08 |
| 15 | ATL | AR | Desaguadero | Río Tunuyán (Dique El Carrizal) (AR) | RTUNY | 8 | -33.3081 | -68.71697 | Sep-06 |
| 16 |  |  |  | Río Atuel (Dique El Nihuil (AR) | RATUE | 36 | -35.0659 | -68.70408 | Oct-06 |
| 17 | ATL | AR | Colorado | Río Colorado (AR) | RCOLO | 28 | -38.2 | -67.4 | Mar-04 |
| 18 | ATL | AR | Limay & Negro | Río Negro (4 Loc) (AR) | RNEGR | 34 | -39.02 | -67.87 | May-04 |
| 19 |  |  |  | Lago Quillén (AR) | LQUIL | 26 | -39.4161 | -71.25 | Jan-98 |
| 20 |  |  |  | Lago Espejo (AR) | LESPE | 29 | -40.6933 | -71.75 | Feb-98 |
| 21 |  |  |  | Lago Morenito (AR) | LMRTO | 30 | 41.06 | 71.53 | Mar-98 |
| 22 | PAC | AR | Puelo | Lago Puelo (AR) | LPUEL | 30 | -42.0934 | -71.61515 | Jan-05 |
| 23 |  |  |  | Lago Inferior (CH) | LINFR | 21 | -42.0924 | -71.79048 | Jan-08 |
| 24 |  |  |  | Lago Azul (CH) | LAZUL | 5 | -41.9501 | -71.85744 | Jan-08 |
| 25 |  |  |  | Lago Tagua Tagua (CH) | LTAGU | 1 | -42.0902 | -72.28794 | Jan-08 |
| 26 |  |  |  | Río Puelo (CH) | RPUE1 | 10 | -42.1692 | -72.41033 | Jan-08 |
| 27 |  |  |  | Lago Candelaria (CH) | LCAND | 6 | -42.2234 | -72.32755 | Jan-08 |
| 28 |  |  |  | Río Puelo (CH) | RPUE2 | 4 | -42.1647 | -72.34241 | Jan-08 |
| 29 | PAC | AR | Futalaufquen/Yelcho | Lago Futalaufquen (AR) | LFUTA | 17 | -42.5 | -71.4 | Jan-00 |
| 30 |  |  |  | Laguna Espejo (CH) | LgESP | 20 | -43.1852 | -71.85953 | Mar-08 |
| 31 | ATL | AR | Chubut | Lago Musters (AR) | LMSTR | 32 | -45.2 | -69.1 | Jan-01 |
| 32 |  | AR | Baker | Estero Burgos (CH) | EBURG | 23 | -47.2473 | -72.53206 | Jan-07 |
|  |  |  |  |  |  |  |  |  |  |
| 33 | PAC | AR |  | Lago Pueyrredón (AR) | LPUEY | 35 | -47.3 | -71.9167 | Jan-01 |
| 34 |  |  |  | Laguna Silvia (CH) | LgSIL | 23 | -46.6998 | -72.48146 | Jan-07 |
| 35 |  |  |  | Río Baker en Balseo Colonia (CH) | RBAK1 | 5 | -47.2029 | -72.63138 | Dec-08 |
| 36 |  |  |  | Laguna Maldonaldo (CH) | LgMAL | 20 | -47.2572 | -72.51634 | Jan-08 |
| 37 |  |  |  | Laguna Esmeralda (CH) | LgESM | 21 | -47.3195 | -72.60738 | Jan-07 |
| 38 |  |  |  | Laguna Juncal (CH) | LgJUN | 2 | -47.3467 | -72.70143 | Jan-08 |
| 39 |  |  |  | Laguna Los Ñadis (CH) | LgÑAD | 1 | -47.707 | -73.16324 | Dec-08 |
| 40 |  |  |  | Río Vargas (CH) | RVARG | 13 | -47.707 | -73.16324 | Dec-08 |
| 41 |  |  |  | Río Baker en Brazo Chico (CH) | RBAK2 | 2 | -47.8226 | -73.44754 | Dec-08 |
| 42 | ATL | AR | Chalia | Río Chico-Chalia (AR) | RCHAL | 33 | -49.6041 | -71.48329 | Jan-07 |
| 43 | PAC | AR | Pascua | Lago San Martin (AR) | LSMRT | 10 | -49.0318 | -72.24448 | Jan-07 |
| 44 | ATL | AR | Santa Cruz | Lago Viedma (AR) | LVDMA | 28 | -49.5738 | -72.34769 | Jan-07 |
| 45 |  |  |  | Lago Argentino (AR) | LARGN | 33 | -50.2 | -72.4 | Jan-01 |
| 46 |  |  |  | Río Santa Cruz (AR) | RSCRZ | **1** | 50 | 72 | Jan-07 |
|  |  |  |  |  |  | **835** |  |  |  |

*: No samples were secured from the headwaters of the Valdivia system (Lago Lácar, Argentina)

Table S2. Motif, number of alleles, and % missing data for each of the 53 markers used.

| Locus Name | Left Primer Sequence | Right Primer Sequence | Motif | Number of Alleles (Entire Dataset) | Average Number of Alleles Per Population | Allele Size Range (Entire Dataset) |
| --- | --- | --- | --- | --- | --- | --- |
| Ptr1 | CCTCGATGAATAGTCTCTACGC | GCCAAGCATCTGACACCAAG | AC | 8 | 3.696 | 18-32 |
| Ptr3 | ATGTTCCAGTCCACCCAACC | TATGAGTGCTTGTGGGTGCC | AC | 21 | 3.391 | 19-65 |
| Ptr4 | TCTCCTGCCGTTTCTCAATAC | AGGACAGGAAAGAGGGTGTG | AC | 10 | 2.348 | 14-34 |
| Ptr5 | TGACTGACTGACTTGCTCTCC | GCGCTTAATTCAACACTGACC | CT | 13 | 2.370 | 25-51 |
| Ptr10 | ACCTGAGATGAGACGTGGTG | CTGAAGGACTGCTGAACGTG | GT | 16 | 3.130 | 16-56 |
| Ptr13 | AAAGGTAGAAGTGCCCGTG | ATACCAGCTTTGTGTGTCGC | AC | 7 | 2.043 | 24-36 |
| Ptr14 | TCTTCTTATTTCCAGCAGCAGG | ACATGCTGACCTCGCCTC | GT | 10 | 2.283 | 25-57 |
| Ptr15 | TGAAATGAGAAGTGGCAGTCC | ATCTTACACACAGGCCCGTC | GT | 11 | 2.500 | 18-46 |
| Ptr16 | GCCAAACCCTGCCTGAAC | ACCAATATGCGTCACAACCC | AC | 7 | 1.848 | 22-44 |
| Ptr18 | GCCATCGTACTGAACACCAC | CACCAGGCTTCATGGTAGTG | AC | 27 | 4.478 | 21-55 |
| Ptr20 | CGCTTGCACATCTAGACAGTC | CAACAGCAGCTCAGGGTTAC | GAT | 7 | 3.087 | 17-35 |
| Ptr21 | CTCTGATTACCACTGTGCTGC | TCGCATATATTTCACAGCCACC | GAT | 6 | 2.804 | 18-33 |
| Ptr24 | TGGAGAAGTGTAACATGCGC | CTCTGCTGGGTGGTGTATGG | AC | 10 | 2.587 | 20-58 |
| Ptr28 | TCAGTCAAGGTTCCCACAGC | CAGGACCAGGATGCCATCAG | GT | 23 | 4.043 | 32-84 |
| Ptr29 | CTTGTTGAGGTAGTGGAGTGC | TGAAGGGTCAGTTCGATTGC | AC | 7 | 2.304 | 28-40 |
| Ptr30 | TGGCTCAGGTGACATTTGTG | CAACAGCAGTCCGTGTTCAG | GT | 6 | 1.717 | 31-41 |
| Ptr32 | TCCCACTTAGACCCAGCAAG | ATCTGCCACTCATCTCCACC | AG | 9 | 3.130 | 28-42 |
| Ptr34 | CCACAGCAGGAAGTATGAGTG | CACATCAGCGTTACATCATAGC | GT | 22 | 3.000 | 11-17 |
| Ptr35 | GGGTTGTGGATCTCTTGCTC | GGAGTGTAGAGGCTGGAGTG | GT | 4 | 1.870 | 38-46 |
| Ptr36 | ACCTACTTTCACAGAGTCCCAC | GCAGCACCAGATAAACAGCC | GT | 14 | 2.087 | 28-60 |
| Ptr37 | AAATGCACCAGTATGAGCGC | TTGTGTAGCAGGCATGTGTG | AC | 15 | 3.370 | 31-67 |
| Ptr38 | AACACCGCCTCCTGTAGTG | TCCCAGATACGAACGCTCTC | GT | 7 | 2.304 | 32-46 |
| Ptr39 | CATACACCGGCACAATCCAC | AATGTCACAGAGCAGGCTTG | AC | 12 | 4.391 | 24-46 |
| Ptr40 | CATCAATGCTGCACTCCATG | TGCCACATCATCACTGTGAC | GT | 11 | 2.435 | 38-64 |
| Ptr42 | ACGTCAACATTAGCACAGCC | CGAGCTGTTGTCTTGCATCG | AGC | 5 | 1.804 | 30-42 |
| Ptr43 | CGTGTGGTTCAGCATTAGGG | TGGCACATCAGTTTCATGGC | AC | 29 | 5.739 | 25-83 |
| Ptr45 | CATTTGCCTCATCCTCACTACC | AGCTGTGGTCAATATGCAGG | ATC | 9 | 2.435 | 23-47 |
| Ptr46 | TTGGCAGCAGAGATCGACAG | TTCACCTCAAACGCTTCAGG | ATC | 11 | 2.261 | 28-58 |
| Ptr47 | TGAATCATTTGCACAACTACCG | TGTGTCATGGAGAGCTGTTG | AGC | 8 | 1.935 | 23-47 |
| Ptr49 | GAGGAATCTTACACCGGCTG | GTGTCACTCGCCCACTGTAG | GT | 9 | 2.957 | 32-54 |
| Ptr50 | GAGTTCGATTGCAGACAGGC | CACTTTCACTCACGTACACTCC | GT | 5 | 2.783 | 36-44 |
| Ptr51 | CTGCCACGAGTAGACAGCC | TGTTCCCATACAGCTCTCAGAG | GCT | 4 | 1.935 | 31-46 |
| Ptr52 | AAGACTCCGCAGATCATCCG | GTACCCGTCAATGCAAACCC | GCT | 6 | 1.935 | 35-50 |
| Ptr54 | GTGTCAGTCTTCAACATCCCTG | GGAAGAGATCACCCGTGGAG | ATC | 6 | 2.478 | 30-48 |
| Ptr55 | AGCAAGTCAGATCCACCCAC | GTGTGAGTCTAGGTTTGAGCAG | AAC | 6 | 2.087 | 37-55 |
| Ptr56 | GCCTGAATGCATCCTGAGTAG | TGCAGGAGTAACTAACAAGGTG | GCT | 11 | 3.978 | 33-63 |
| Ptr57 | GTTCTGAAATCGCTGAGAGGAC | ACCTGTGTGATAAACGTCCG | GAT | 8 | 1.674 | 34-55 |
| Ptr58 | GTGTGAGTCTAGGTTTGAGCAG | AGCAAGTCAGATCCACCCAC | GTT | 6 | 2.065 | 37-55 |
| Ptr59 | CAGGAAGCGTATGTGGTGAG | TTTCCCTCTCCCAACCTGC | GAT | 8 | 2.913 | 34-52 |
| Ptr60 | CTCACACACCACTTACCGTC | CCTCGGTCGCCATCTTGAC | ACC | 7 | 2.587 | 34-52 |
| Ptr61 | TCCTCCCTCCTCAGCAGC | GAAGGACAAAGGAGAGACTGC | CTT | 4 | 1.109 | 43-52 |
| Ptr62 | CGGCTTCTGTTAGACATTCAGC | GAGGAGTTGTGTGTCAGTCAG | ATC | 8 | 1.804 | 37-61 |
| Ptr64 | ATCCATGCTGTTTGTTCCCG | TGCACCATACCGTTGTTTGC | CTT | 18 | 6.087 | 42-90 |
| Ptr65 | TGCTGTCGTGCCTACTCAAC | GGACACCACTGTTTGCTGC | CTT | 5 | 2.326 | 43-58 |
| Ptr66 | GTGGACGGTCTTGGTGGTC | AGGGTTGAGCTCCTTCTTCC | AGG | 9 | 3.022 | 40-64 |
| Ptr67 | CCATTGTCAGCCATGTGAGG | GACGTTCATGGTGTTAGGCC | CCT | 10 | 3.239 | 33-66 |
| Ptr69 | GGCAGGCAGATAAAGCAAGG | AACAGTGATGAAGAGGTGTACC | AGC | 11 | 2.196 | 37-88 |
| Ptr70 | TAGCATAAGAGTGGGCGGAG | GTAGGCTGGGTCTGATCTCC | CT | 3 | 1.152 | 52-64 |
| Ptr71 | TATACATCCGTCGCCTGAGC | TCCGACAATTTAGCGTAGACTG | AC | 18 | 4.109 | 39-60 |
| Ptr72 | GGCATTGAGTCTGGTTGAGC | AAGAGTCCGACTGAGCTGTG | ATC | 6 | 2.152 | 40-58 |
| Ptr73 | CCCTCTCTGTGTCCTTCCAG | CATGCCTGTCACAACACGG | ACC | 11 | 3.348 | 40-56 |
| Ptr74 | CATCGTGGTTTCAGTGAGGC | AGTGGAGGGAAGATGTGAGC | AGC | 5 | 1.370 | 40-55 |
| Ptr75 | TGGATTGATGAGCTTGCACAG | ACGATAATGAGGCTGTTTGTGG | ATC | 10 | 2.261 | 34-73 |

Table S3. Hierarchical analysis of molecular variance (AMOVA) with drainages grouped by A) contemporary drainage direction (Atlantic vs. Pacific drainage), and B) ancestral drainage direction in which trans-Andean systems (i.e., systems with headwaters east of the highest Andean peaks but currently draining into the Pacific Ocean) are grouped with Atlantic draining systems. The percentage of variation explained by variation among groups when the groups are defined by contemporary drainage basin (A) is over five times lower than when rivers are grouped by contemporary drainage basin (B). Conversely, the percentage of variance explained by

**a)**

| **Source of Variation** | **Df** | **Sum of Squares** | **Variance of Components** | **% variation** |
| --- | --- | --- | --- | --- |
| **Among Groups** | 1 | 878.93 | 0.75 | **5.00** |
| **Among Populations Within Groups** | 44 | 8481.42 | 5.16 | 34.63 |
| **Among Individuals Within Populations** | 789 | 7504.95 | 0.53 | 3.54 |
| **Within Individuals** | 835 | 7062.50 | 8.46 | 56.82 |
| **Total** | 1669 | 23927.81 | 14.9 |  |

**b)**

| **Source of Variation** | **Df** | **Sum of Squares** | **Variance of Components** | **% variation** |
| --- | --- | --- | --- | --- |
| **Among Groups** | 1 | 3402.51 | 4.62 | **26.94** |
| **Among Populations Within Groups** | 44 | 5957.84 | 3.53 | 20.60 |
| **Among Individuals Within Populations** | 789 | 7504.95 | 0.53 | 3.08 |
| **Within Individuals** | 835 | 7062.50 | 8.46 | 49.38 |
| **Total** | 1669 | 23927.8 | 17.14 |  |

**(c)** Ancestral PUELO considered Pacific drainage

| **Source of Variation** | **Df** | **Sum of Squares** | **Variance of Components** | **% variation** |
| --- | --- | --- | --- | --- |
| **Among Groups** | 1 | 2154.34 | 2.43 | **15.45** |
| **Among Populations Within Groups** | 44 | 7206.02 | 4.33 | 27.47 |
| **Among Individuals Within Populations** | 789 | 7504.95 | 0.53 | 3.35 |
| **Within Individuals** | 835 | 7062.50 | 8.46 | 53.73 |
| **Total** | 1669 | 23927.8 | 15.75 |  |

(d) Ancestral FUTALAUFQUEN/YELCHO considered Pacific drainage

| **Source of Variation** | **Df** | **Sum of Squares** | **Variance of Components** | **% variation** |
| --- | --- | --- | --- | --- |
| **Among Groups** | 1 | 2880.12 | 3.57 | **21.75** |
| **Among Populations Within Groups** | 44 | 6480.24 | 3.87 | 23.52 |
| **Among Individuals Within Populations** | 789 | 7504.95 | 0.53 | 3.21 |
| **Within Individuals** | 835 | 7062.50 | 8.46 | 51.52 |
| **Total** | 1669 | 23927.8 | 16.43 |  |

(e) Ancestral BAKER considered Pacific drainage

| **Source of Variation** | **Df** | **Sum of Squares** | **Variance of Components** | **% variation** |
| --- | --- | --- | --- | --- |
| **Among Groups** | 1 | 1758.65 | 1.82 | **11.85** |
| **Among Populations Within Groups** | 44 | 7601.70 | 4.58 | 29.76 |
| **Among Individuals Within Populations** | 789 | 7504.95 | 0.53 | 3.42 |
| **Within Individuals** | 835 | 7062.50 | 8.46 | 54.96 |
| **Total** | 1669 | 23927.8 | 15.39 |  |

(f) Ancestral PASCUA (lake San Martín/O’Higgins) considered Pacific drainage

| **Source of Variation** | **Df** | **Sum of Squares** | **Variance of Components** | **% variation** |
| --- | --- | --- | --- | --- |
| **Among Groups** | 1 | 3204.81 | 4.23 | **25.08** |
| **Among Populations Within Groups** | 44 | 6155.54 | 3.65 | 21.66 |
| **Among Individuals Within Populations** | 789 | 7504.95 | 0.53 | 3.12 |
| **Within Individuals** | 835 | 7062.50 | 8.46 | 50.14 |
| **Total** | 1669 | 23927.8 | 16.87 |  |

Seven collections have sample sizes N≤2. These collections are (See Table 1):

Biobío system

(5) Ramadillas (RRAMD) (N=1)

Valdivia system

(6) Rio Cruces (RCRUC) (N=2)

Puelo system

(25) Tagua Tagua lake (LTAGU) (N=1)

Baker system

(38) Lag Juncal (LgJUN) (N=2)

(39) Lag. Los Ñadis (LgÑAD) (N=1)

(41) Rio Baker (RBAK2) (N=2)

Santa Crus river system

(46) Sta Cruz river (RSCRUZ) (N=1)

A close examination of fig 2 reveals that in virtually all instances these collections cluster within their corresponding systems. There is one potential exception (RCRUC) which though in the same branch as the other collections from the Valdivia system, clusters closer to the samples from the neighboring rivers to the south, which as RCRUC, are coastal rivers and are geographically quite close (See inset for samples 6 and 9 in Fig.1). These results suggest the small sample sizes of these collections did not affect or bias the overall phylogeographic patterns.
